# CBCT evaluation of inter- and intra-fraction motions during prostate stereotactic body radiotherapy: a technical note

**DOI:** 10.1186/s13014-020-01534-2

**Published:** 2020-04-19

**Authors:** Omar Jmour, Marouan Benna, Pierre Champagnol, Majed Ben Mrad, Anis Hamrouni, Layal Obeid, Chaimaa Lahmamssi, Amal Bousarsar, Nicolas Vial, Amel Rehailia-Blanchard, Sandrine Sotton, Meiling Lan, Julien Langrand-Escure, Alexis Vallard, Nicolas Magné

**Affiliations:** Department of radiation oncology, Lucien Neuwirth Cancer Institute, 108 Bis, Avenue Albert Raimond, 42270 Saint Priest en Jarez, France

**Keywords:** Prostate cancer, Radiotherapy, Stereotactic body radiotherapy, Cone beam CT, motion, Bladder, Rectum, Organs at risk

## Abstract

**Background:**

In most clinical trials, gold fiducial markers are implanted in the prostate to tune the table position before each radiation beam. Yet, it is unclear if a cone-beam computed tomography (CBCT) should be performed before each beam to monitor a possible variation of the organs at risk (OARs) fullness, especially in case of recto-prostatic spacer implantation. The present study aimed at assessing the inter- and intra-fraction movements of prostate, bladder and rectum in patients implanted with a hyaluronic acid spacer and undergoing prostate stereotactic body radiotherapy (SBRT).

**Methods:**

Data about consecutive patients undergoing prostate SBRT were prospectively collected between 2015 and 2019. Inter-and intra-fraction prostate displacements and volume variation of organs at risk (OARs) were assessed with CBCTs.

**Results:**

Eight patients were included. They underwent prostate SBRT (37.5Gy, 5 fractions of 7.5Gy) guided by prostate gold fiducial markers. Inter-fraction variation of the bladder volume was insignificant. Intra-fraction mean increase of the bladder volume was modest (29 cc) but significant (*p* < 0.001). Both inter- and intra-fraction variations of the rectum volume were insignificant but for one patient. He had no rectal toxicity. The magnitude of table displacement necessary to match the prostate gold fiducial marker frequently exceeded the CTV/PTV margins (0.4 cm) before the first (35%) and the second arc (15%). Inter- and intra-fraction bladder and rectum volume variations did not correlate with prostate displacement.

**Conclusion:**

Major prostate position variations were reported. In-room kV fiducial imaging before each arc seems mandatory. Intra-fraction imaging of the OARs appears unnecessary. We suggest that only one CBCT is needed before the first arc.

**Trial registration:**

NCT02361515, February 11th, 2015.

## Background

Recent phase 2 and 3 trials [1, 2] tend to show that utrahypofractionation (≥5Gy per fraction) is the next step in the evolution of prostate cancer external beam radiotherapy [1, 2].

However, such high doses *per* fraction require a specific management of the inter- and intra-fraction movements of the target. In clinical trials, gold fiducial markers were implanted in the prostate to tune the table position before each radiation beam. Yet, it is unclear if a cone-beam computed tomography (CBCT) should be performed before each beam to monitor a possible variation of the organs at risk (OARs) fullness [3–5], especially when performed with hyaluronic acid spacer. The present technical note was designed to assess this question.

## Methods and materials

Data about consecutive patients undergoing a curative prostate SBRT at the Lucien Neuwirth cancer center (France) were prospectively collected between 2015 and 2019.

### Procedure

All patients had gold fiducial markers implanted in the prostate. A hyaluronic acid spacer was placed between the anterior rectal wall and the posterior face of the prostate. The CTV was defined as the prostate only on the CT images coupled with a T2w-MRI. The CTV–PTV margin was 0.4 cm in every direction. The rectum was delineated 2 cm above and under the PTV. The bladder was delineated entirely. Dose constraints were as follows. For the rectum: V37.5Gy < 3 cc, V25Gy < 10 cc, V39.5Gy < 1 cc. For the bladder: V39.5Gy < 1 cc. For the “bladder-PTV”: V30Gy < 10 cc. Patients were treated with the RapidArc® technology based on 2 consecutive arcs and received 37.5Gy in 5 fractions (beam energy: 6MV) with at least 48 h between each fraction. Patients were treated on a Novalis Truebeam (Varian, California, USA) and immobilized with a knee-immobilization device on a robotic 6-degree couch. They were asked to empty their bowel and bladder 60 min before each radiation course and then, to drink 350-500 cc. Before each arc, a CBCT was performed and a radiation oncologist decided which table displacements should be achieved to match the fiducial markers. Prostate and OARs were then contoured on the 2 CBCTs by one operator (2nd author, the same operator who contoured the planning CT).

### Statistical analysis

Pearson’s correlation and Student tests were performed to compare prostate motion (i.e. table displacements) distributions. The paired student t-test was used to compare variations of OARs volume. The Fisher exact test was used to asses if toxicity and OARs fullness were linked. Significance was defined as *p* < 0.05.

## Results

### Patients and treatment characteristics

Eight patients were included. The mean duration of a fraction was 28 min (range: 13-62 min). No patient experienced biochemical or clinical failure during the 33-month median follow-up (range: 17–37). Two patients had acute grade 2 urinary toxicity (retention requiring a urinary catheter). There was no grade 2 digestive toxicity or acute grade > 2 toxicity. Two patients experienced late grade 2 urinary toxicity (dysuria, urinary incontinence), one experienced late grade 3 urinary toxicity (incontinence) and one experienced late grade 2 digestive toxicity (rectal bleeding). There was no late grade > 3 toxicity.

### Fiducial marker guided prostate radiotherapy

- *Inter-fraction assessment****(***Table 1***)***

**Table 1:** Magnitude of table displacement performed after the CBCTs in order to target the prostate (matching on gold fiducials)

	CBCT performed before the first arc			CBCT performed before the second arc		
	Antero-posterior	Supero-inferior	Left-Right	Antero-posterior	Supero-inferior	Left-Right
Minimum value (cm)	−0.89	− 0.60	−1.96	−1.80	− 0.37	− 0.40
Maximum value (cm)	+ 0.88	+ 0.45	+ 0.51	+ 1.64	+ 0.28	+ 1.79
Mean value± Standard deviation (cm)	0.06 ± 0.35	−0.04 ± 0.26	0.00 ± 0.27	−0.03 ± 0.34	−0.07 ± 0.13	0.04 ± 0.23
Interval including 95.4% of values (cm)						
Lower boundary	−0.63	−0.56	− 0.55	−0.70	− 0.32	−0.42
Upper boundary	+ 0.76	+ 0.49	+ 0.55	+ 0.65	+ 0.19	+ 0.51
Proportion of table displacement exceeding the CTV/PTV margin (0.4 cm), %	35%	30%	17.5%	12.5%	5%	15%

The mean table displacement before the first arc was 0.06 cm in the antero-posterior direction (standard deviation (SD) = 0.35 cm, range: − 0.89 cm to + 0.88 cm), − 0,04 cm in the supero-inferior direction (SD = 0.26 cm, range: − 0.60 cm to + 0.45 cm), and 0.00 cm in the left-right direction (SD = 0.27 cm, range: − 1.96 cm to + 0.51 cm).

b. *Intra-fraction assessment****(***Table 1***)***

The mean table displacement before the second arc was − 0.03 cm in the antero-posterior direction (SD = 0.34 cm, range: − 1.80 cm to + 1.64 cm), − 0,07 cm in the supero-inferior direction (SD = 0.13 cm, range: − 0.37 cm to + 0.28 cm) and 0.04 cm in the left-right direction (SD = 0.23 cm, range: − 0.40 cm to + 1.79 cm).

### Variation of OARs volume

**Fig. 1:**
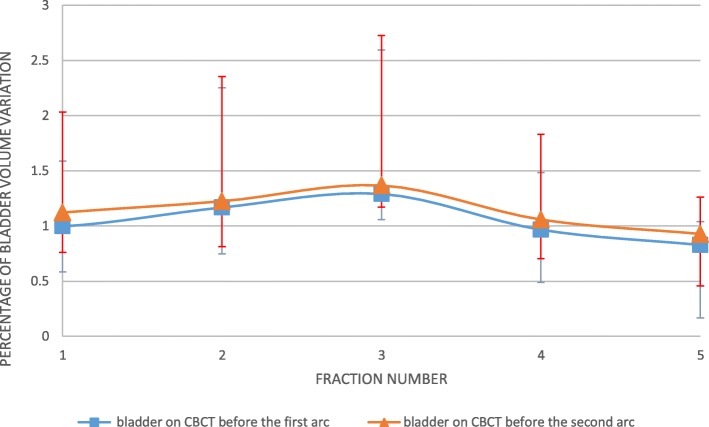
Evolution of the mean bladder volume before and during SBRT fraction, compared to the bladder on planning CT-scan. The bladder volume assessed on the planning CT-scan divided the bladder volume assessed on the CBCT performed before the first (blue line) and the second arc (orange line) (1 = volumes were equal)

1. *Bladder*
  *1.a. Inter-fraction assessment****(***Fig. 1***).***

The mean bladder volume on the planning CT-scan was 368 cc (range:161-607 cc). Three patients had a mean bladder volume during SBRT (calculated on the CBCT performed before each of the five fractions) inferior to the bladder volume on the planning CT-scan (range: − 10% to − 29%).

When the whole set of patients was taken into account, there was no statistically significant inter-fraction variation of the mean bladder volume (*p* = 0.12). There was no correlation between bladder fullness and the inter-fraction prostate movement (i.e. table displacement performed before the first arc) -whatever the considered direction (antero-posterior: r^2^ = 0.31; supero-inferior: r^2^ = 0.16; left-right: r^2^ = − 0.38).

*1.b. Intra-fraction assessment.*

**Fig. 2:**
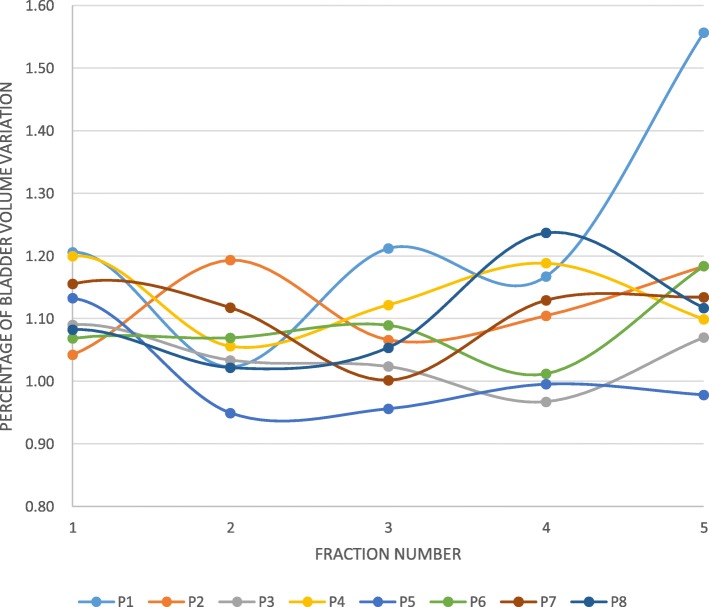
Individual data about the evolution of the bladder volume assessed during SBRT fraction, compared to the bladder assessed before SBRT fraction. The bladder volume assessed on the CBCT performed before the first arc divided the bladder volume assessed on the CBCT performed before the second arc (1 = volumes were equal). Abbreviation: P: Patient

The mean bladder volume significantly increased between the first and the second arc (mean increase: 29 cc *p* < 0.001, Fig. 1). For four patients, the volume increased by 20%, at least once. Two patients experienced urinary leakage at least once and a decrease of bladder volume on the second CBCT (Fig. 2). There was no correlation between bladder fullness (assessed before the second arc) and the intra-fraction prostate movement (i.e. table displacement performed before the second arc) -whatever the considered direction (antero-posterior: r^2^ = − 0.02; supero-inferior: r^2^ = − 0.22; left-right: r^2^ = − 0.24).

**Fig. 3:**
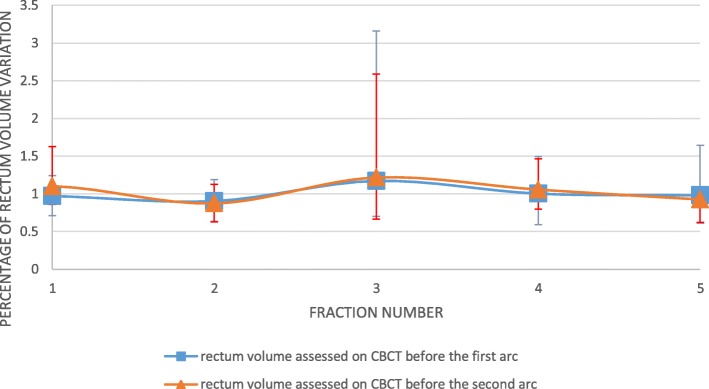
Evolution of the mean rectum volume assessed before and during SBRT fraction, compared to the rectum on planning CT-scan. The rectum volume assessed on the planning CT-scan divided the rectum volume assessed on the CBCT performed before the first (blue line) and the second arc (orange line) (1 = volumes were equal)

2. *.Rectum.*
  *1.a. Inter-fraction assessment****(***Fig. 3***).***

The mean rectum volume on the planning-CT was 66 cc (range:38–108.5 cc). The mean rectum volume assessed before the first arc did not significantly vary compared to the mean volume assessed on the planning-CT scan (*p* = 0.40). There was no significant variation of inter-fraction rectal volume. There was no correlation between rectum fullness and the inter-fraction prostate movement whatever the considered direction (antero-posterior: r^2^ = 0.10; supero-inferior: r^2^ = 0.006; left-right: r^2^ = 0.07).

*1.b. Intra-fraction assessment.*

**Fig. 4:**
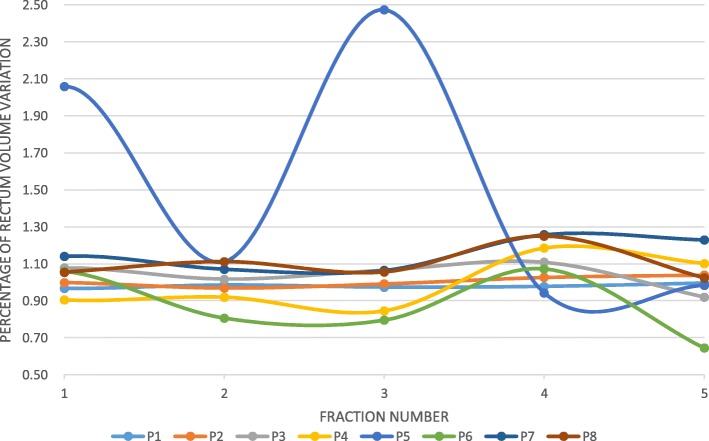
Individual data about the evolution of the rectal volume assessed during SBRT fraction, compared to the rectum assessed before SBRT fraction. The rectal volume assessed on the CBCT performed before the first arc divided the rectal volume assessed on the CBCT performed before the second arc (1 = volumes were equal). Abbreviation: P: Patient

There was no significant variation of the mean rectum volume on the CBCT performed before the second arc when it was compared to the rectum on the CBCT performed before the first arc (*p* = 0.76, Fig. 3). For four patients, the variation reached 20% of the volume assessed on the first CBCT at least once. For one patient, the rectal volume variation exceeded 200% twice (Fig. 4). Intra-fraction variation of rectum fullness and intra-fraction prostate movement did not correlate -whatever the considered direction (antero-posterior: r^2^ = 0.07; supero-inferior: r^2^ = − 0.21; left-right: r^2^ = 0.40).

### Toxicity

Severe (grade ≥ 2) acute or late toxicity correlated neither with prostate movement nor with inter- or intra-fraction fullness of bladder or rectum. Two patients out of three who had suboptimal filled bladder (mean volume on CBCT inferior to the one on planning-CT) had severe late urinary toxicities. One of the five patients who had optimal filled bladder had severe late urinary toxicities.

## Discussion

The present study reports on the intra- and inter-fraction movements of the target and the OARs during a prostate SBRT with hyaluronic acid spacer. Results are preliminary since they were assessed only through a monocentric experience in 8 patients. A huge amount of paper has been published on this topic for patients who had no hyaluronic acid spacer. Their results will not be discussed. Only three studies previously analyzed the inter- and intra-fractional motion with recto-prostatic spacers. Picardi et al. assessed the overall mean inter-fractional prostate displacements [6]. Displacements > 5 mm were found in 0.8, 12.3, and 6.5% CBCT acquisitions in the left-right, antero-posterior, and supero-inferior directions, respectively versus 10, 0 and 17% in our series. Intrafraction prostate motion was assessed by Juneja et al [7]. 5% of the fractions had mean motion more than 3 mm, even for the hypofractionated courses (mean motion: 0.5–2.7 mm for courses > 3 min). In our series, the intra-fraction prostate movement exceeded 3 mm in at least one axe for 32.5% of CBCTs. Hedrick et al. used the same methodology [8]. Maximal values of intra-fraction prostate motion were below 8 mm and only 2.5% exceeded 5 mm. In our series, the intra-fraction prostate movement exceeded 5 mm in at least one direction in 10%. In conclusion, the inter-and intra-fractions displacement assessed in the present study seems to be slightly superior to the ones reported in literature. However, fraction duration were longer in the present cohort. In our series, the proportion of table displacement exceeding the PTV margin (0.4 cm) only partially decreased after the second CBCT. That confirms that prostate movements during long fraction are significant. A fiducial imaging before each arc is mandatory. However, the magnitude of table displacement with extreme values exceeding 1.6 cm after the second CBCT should question the knee-immobilization device that was used. A vacuum-formable mattress could probably significantly avoid intra-fraction patient movements.

**Fig. 5:**
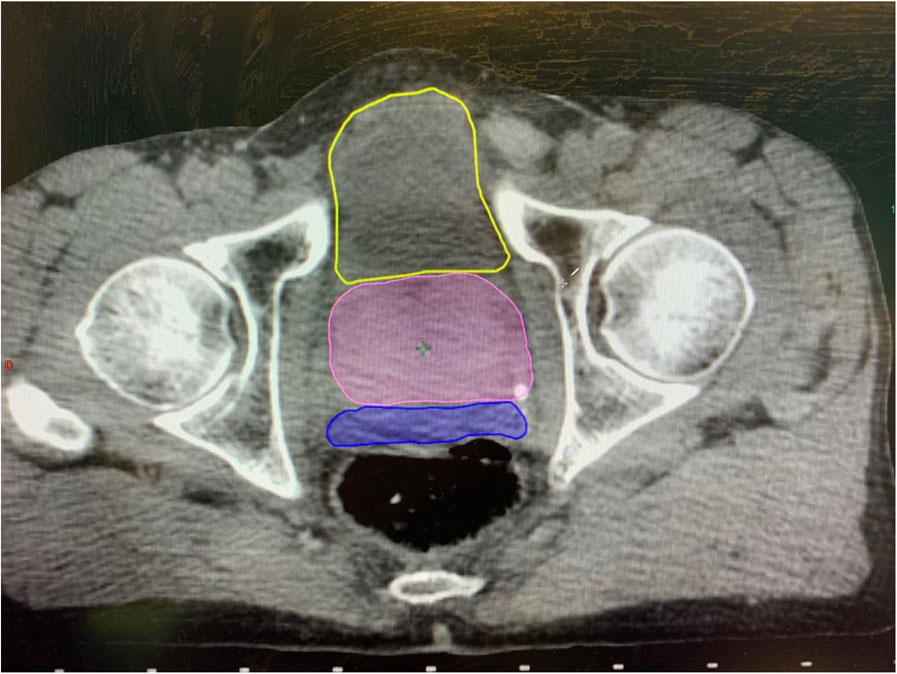
Example of the effect of hydrogel rectal spacer (blue), preventing the rectal wall to move anteriorly. The prostate is in pink, the bladder in yellow

Regarding the bladder, there was no significant inter-fraction fullness variation. Urinary toxicities were not correlated with the inter- or intra-fraction variation of the bladder volume. However, 2 out of the 3 patients who had suboptimal bladder fullness experienced severe late toxicity. This should probably call for a systematic CBCT assessment before each fraction to check the bladder is at least as full as it was on the planning-CT scan. No significant data was brought by the second CBCT (performed before the second arc). Regarding the rectum, there was no significant inter-fraction fullness variation. There was no major intra-fraction volume variation but for one patient who experienced no toxicity. This was expected since all patients had a hyaluronic acid spacer that prevented the rectal wall to move anteriorly [9] (Fig. 5). Therefore, thanks to rectal spacer, monitoring the variation of the rectal volume during SBRT fraction turns unnecessary. No significant data was given by the CBCT performed before the second arc. Thus, intra-fraction imaging of the OARs appears unnecessary.

## Conclusion

Fiducial imaging (kV/kV) before each arc seems mandatory whereas intra-fraction imaging of the OARs appears unnecessary. We suggest that only one CBCT is needed before the first arc.

## Data Availability

The datasets used and/or analysed during the current study are available from the corresponding author on reasonable request.

## Abbreviations

CBCT: Cone-beam computed tomography
OAR: Organs at risk
kV: KiloVolt
SBRT: Stereotactic body radiotherapy
CTV: Clinical target volume
PTV: Planning target volume
